# A tunable physiomimetic stretch system evaluated with precision cut lung slices and recellularized human lung scaffolds

**DOI:** 10.3389/fbioe.2022.995460

**Published:** 2022-10-03

**Authors:** Oskar Rosmark, Arturo Ibáñez-Fonseca, Johan Thorsson, Göran Dellgren, Oskar Hallgren, Anna-Karin Larsson Callerfelt, Linda Elowsson, Gunilla Westergren-Thorsson

**Affiliations:** ^1^ Lung Biology, Department of Experimental Medical Science, Lund University, Lund, Sweden; ^2^ Transplant Institute and Department of Cardiothoracic Surgery, Sahlgrenska University Hospital, Gothenburg, Sweden; ^3^ Division of Thoracic Surgery, Department of Clinical Sciences, Lund University, Lund, Sweden

**Keywords:** mechanical stimuli, lung, epithelial cells, extracellular matrix, stretch, 3D, precision cut lung slice

## Abstract

Breathing exposes lung cells to continual mechanical stimuli, which is part of the microenvironmental signals directing cellular functions together with the extracellular matrix (ECM). Therefore, developing systems that incorporate both stimuli is urgent to fully understand cell behavior. This study aims to introduce a novel *in vitro* culture methodology combining a cyclic stretch that simulates *in vivo* breathing with 3D cell culture platforms in the form of decellularized lung slices (DLS) and precision cut lung slices (PCLS). To this end, we have constructed a device that mimics the amplitudes and frequencies of distensions seen in the breathing human lung. For its validation, we cultured H441 lung epithelial cells in human DLS exposed to 16 stretch cycles per minute with a 10% stretch amplitude. Cell viability (resazurin reduction), proliferation (Ki-67) and YAP1 activation were evaluated at 24 and 96 h by immunohistochemistry, while the expression of SFTPB, COL3A1, COL4A3 and LAMA5 was evaluated by qPCR. Cyclic stretch induced an increase in SFTPB expression after 24 h without a concomitant increase in the stretch responsive gene YAP1. Moreover, the ECM milieu lowered the expression of the basement membrane protein genes COL4A3 and LAMA5 compared to tissue culture plastic control cultures, but no effect was observed by the mechanical stimuli. The device also confirmed good compatibility with PCLS culture, showing preserved morphology and metabolism in rat PCLS after 72 h of mechanical stretch. Thus, we present a novel device and methodology for the easy assembling and study of lung tissue slice cultures subjected to physiomimetic mechanical stimuli, which shows promise for future studies of cell and tissue function in a lung ECM milieu with physiological or pathological mechanical stimuli.

## Introduction

Parenchymal lung cells are exposed to mechanical stretching already before birth. This stimulus is crucial for normal lung development and lung cells remain responsive to this stretch throughout life, both in homeostasis and in pathological settings (Liu et al., 1999; Schmitt et al., 2012; Schittny, 2017). For instance, excessive mechanical stimuli in the setting of ventilator-induced lung injury, trigger numerous cellular responses that may lead to extracellular matrix (ECM) remodeling (Pelosi and Rocco, 2008). Uncontrolled remodeling is also a key feature of lung fibrosis, where the initiation and progression of the most common presentation, idiopathic pulmonary fibrosis (IPF), follows the distribution of the mechanical forces in the lung parenchyma (Leslie, 2012; Carloni et al., 2013; Wu et al., 2020).

Important lessons regarding the biological and physiological effects of mechanical forces have been learned using *in vivo* animal models, but *in vitro* models capturing some of the crucial aspects of the *in vivo* conditions are needed to facilitate high throughput studies and circumventing issues with species differences between animal models and humans (Schmitt et al., 2012). There are several *in vitro* studies of epithelial and mesenchymal lung cells cultured under uniaxial or biaxial strain, however, few incorporate the complex ECM microenvironment found in the lung (Chess et al., 2000; Novak et al., 2021). We and others have shown the fundamental impact of the lung ECM microenvironment on cellular behavior and phenotype (Parker et al., 2014; Elowsson Rendin et al., 2019; Hedström et al., 2021). Of specific interest is the composition and integrity of the basement membrane composed largely of laminins and collagen type IV and its interaction with epithelial cells (Timpl, 1989; Pierce et al., 1998; Pelosi and Rocco, 2008). Also, the production of surfactant protein B (SFTPB) due to its role in lowering surface tension for increased gas exchange, which is believed to depend on a functional epithelial layer (Wert et al., 2009; Fehrholz et al., 2017).

Precision cut lung slices (PCLS) have proven to be a valuable tool to facilitate high-throughput studies of both basic lung biology as well as drug and toxicology screening. The PCLS platform allows the study of lung tissue with its wide array of cell types *ex vivo*, however, it is hard to maintain live cultures and sustained functionality for more than a few days and the inherent complexity of PCLS present a challenge when trying to understand mechanisms at a cellular level (Liu et al., 2019). For this reason, we and other groups have successfully used decellularized lung slices (DLS) as a more practical platform with less complexity while still providing a complex 3D environment where cells can interact with tissue specific ECM structures (O'Neill et al., 2013; Rosmark et al., 2018).

In this work, we take the *in vitro* use of DLS one step further towards an *in vivo*-like setting by introducing tensile mechanical stimuli while still maintaining the potential for high throughput studies. Specifically, we have developed a device for cyclic stretching of lung slices with programmable amplitude and frequency of the movement, which was experimentally characterized. Moreover, we have used this device to study the effect of cyclic stretch on the human lung epithelial cell line H441, cultured in decellularized human lung slices as well as on naïve PCLS. Our readouts focus on the expression of key ECM components, hypothesizing that the stretch would induce an altered synthesis profile.

## Material and methods

### In-house stretch device

**FIGURE 1:**
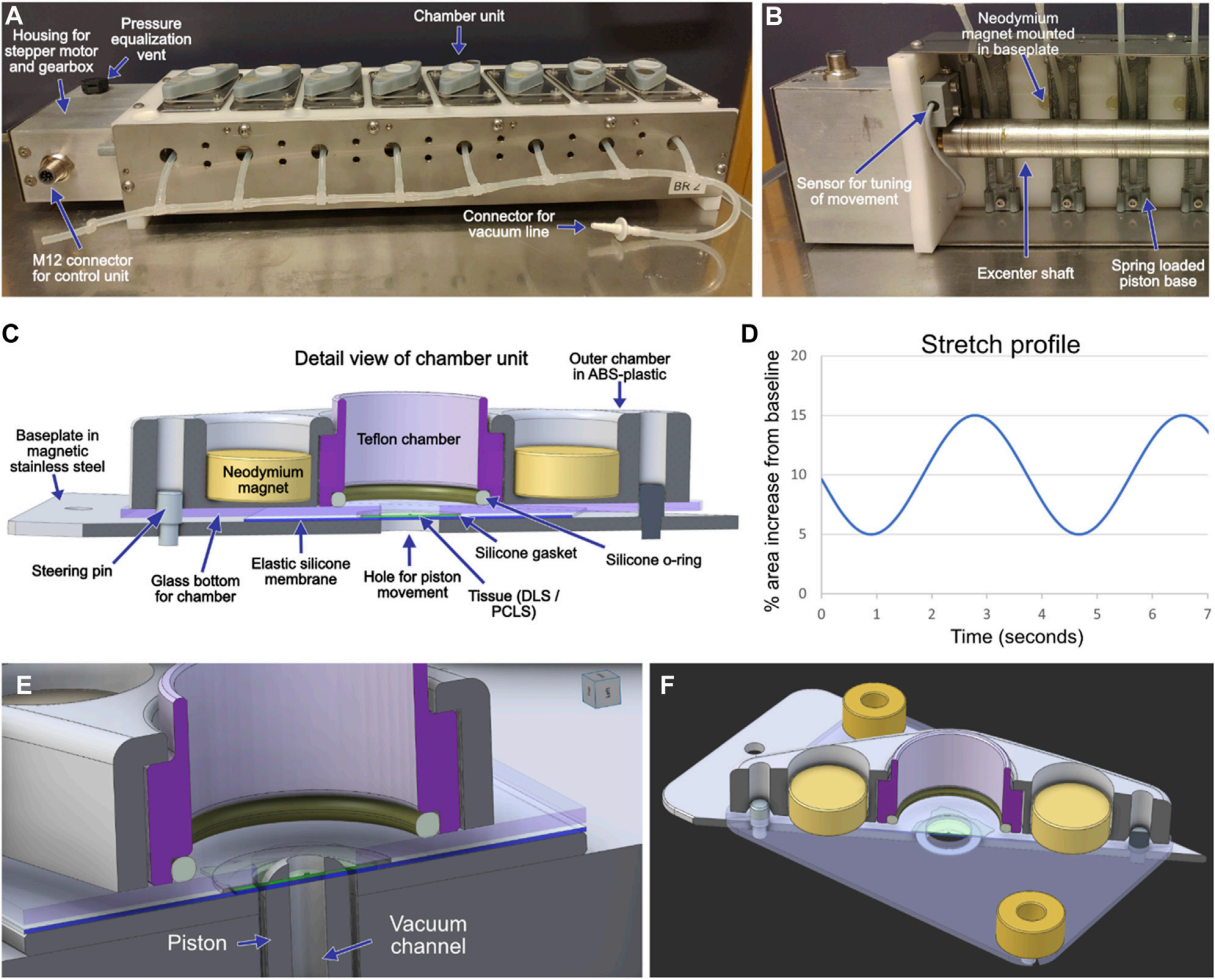
An image of a stretch device with eight chamber units for mounting of lung slices **(A)**. The unit is placed in a conventional tissue culture incubator and controlled by a programmable control unit placed outside the incubator. A piston is located underneath each chamber unit and produces the stretch movement by pushing the elastic membrane and the lung slice upwards. A vacuum line is connected to ensure contact between the elastic membranes and the driving pistons during the return phase. An image of part of the underside of a stretch device shows the off-center mounted shaft that drives the movement of the piston **(B)**. A sensor feeds information on the achieved movement to the control unit as part of a closed loop-control system. A schematic outline of the design of the chamber units **(C)**. Each chamber unit is held together by the attraction between the neodymium magnets and the metallic baseplate. Additional magnets mounted in the baseplate **(B)** help keep the chambers together when mounted on the stretch device. The movement of the shaft and thereby the achieved stretch can be programmed to emulate different breathing patterns, a simple sinusoidal profile has been used in the current study **(D)**. Close-ups of the well design **(E,F)**.

The basic construction of the stretch device is made up of a stationary platform with 8 mounted chambers (Figures 1A,B). Along the centre of the platform a rotating excentre shaft of stainless steel, driven by a stepper motor, creates vertical movement of 8 pistons that push upwards on elastic silicone membranes at the bottom of each chamber, on which the DLS/PCLS are placed. Each Ø6 mm piston, with an isosceles trapezoid shape and slightly convex, moves through a Ø7 mm hole in the baseplate and the bottom of each chamber, and just reaches the elastic membrane at its bottom position. It therefore pushes the membrane and the tissue homogeneously in most of its extension, excluding the edges. The stepper motor is programmed to rotate the off-centre shaft back and forth, thereby displacing the elastic membrane upward, creating an increase in the surface area of 0%–30%. The area expansion is estimated using the assumption that the stretched elastic membrane has a shape similar to a spherical cap, calculating the area of the cap with the formula:

$$Area=\pi \left(a^{2}+h^{2}\right)$$

Where *a* is the radius of the base of the cap and *h* is the height. The % stretch is then calculated by dividing that number by the area of the circle formed with the piston at its bottom position and multiplying by 100. The tips of the pistons are slightly convex, increasing the accuracy of this approximation. The practically usable maximum frequency is about 40 cycles per minute, even higher frequencies can be achieved by allowing the shaft to rotate continually in the same direction, with the stretch amplitude governed by maximal displacement of the fitted shaft. The pistons are 3D printed in ABS-plastic and have a 1 mm central canal allowing for connection of a vacuum line that ensures adherence of the membrane to the piston during the downward return movement. Each chamber is composed of a bottom plate of magnetic stainless steel with a 0.3 mm thick 60°ShA silicone membrane (approx. 30 × 30 mm) placed over its centre, on which the tissue slice is gently placed and completely spread out (Figures 1A,C). A 1 mm thick and 1.5 mm wide 40°ShA silicone gasket with an Ø7 mm inner diameter is placed between the tissue slice and an overlying 1 mm thick borosilicate glass slide. The culture medium-containing compartment of the chamber is constructed with an inner Teflon part that is secured against the glass slide *via* a 1.5 thick mm silicone O-ring and held in place by an outer part 3D-printed in ABS-plastic with compartments for two neodymium magnets that hold the lung tissue slice and the chamber construct together by attracting the bottom steel plate of each chamber. The well formed by the Teflon insert has a diameter of 16 mm, giving a surface area of approximately 2 cm^2^, to avoid hypoxic conditions due to a high medium column above the tissue. Each well is covered by a lid that allows for gas exchange but limits evaporation in the incubator and contamination when the unit is transferred between the incubator and the laminar flow bench. The gasket and membrane are disposed after each use and new ones are prepared from large sheets of FDA compliant silicone (Rubberstock, the Netherlands). The silicone parts, the Teflon inner well and the glass and stainless-steel plates are sterilized by autoclaving between each use. The whole stretch device is constructed to fit in a standard tissue culture incubator and to withstand the corrosive environment inside. The device communicates *via* cable with a control unit outside the incubator consisting of a stepper motor drive unit and an Arduino computer. The control unit receives signals from a sensor monitoring the movement of the drive shaft and continually modifies the drive signal to the stepper motor to correct any deviations from the programmed movement (Figure 1B). Programming for the movement was done in Visual studio code (version 1.61.0), and the programmed stretch settings for the DLS and PCLS experiments are visualized in Figure 1D. Close-ups of the well design for the stretch device are shown in Figures 1E,F for clarification.

### Patient material

Human lung tissue was received from Sahlgrenska University hospital in Gothenburg. The lung tissue was obtained from organ donors without known lung disease, where the lungs were not eligible for transplantation. Informed written consent was obtained from the donors or next of kin. The use of human material in this study was approved by the Swedish Ethical Committee in Lund (Dnr 2008/413) and all experimental procedures were performed in accordance with the approved guidelines of the ethical committee. Donor characteristics are presented in Supplementary Table S1.

### Decellularization of human lung slices

DLS were produced as previously described (Rosmark et al., 2018). In short, snap frozen 1 cm^3^ cubes of pleura-adjacent lung tissue were cryosectioned to a thickness of 350 μm, decellularized by a 4-hour exposure to serial changes of a detergent solution containing 8 mM 3-((3-cholamidopropyl) dimethylammonio)-1-propanesulfonate (CHAPS), 1 M NaCl and 25 mM EDTA in phosphate buffered saline (PBS) at pH 8.0. Lastly, slices were treated with 90 U/ml of turbonuclease (Sigma-Aldrich, MO, United States), before repeated rinses with PBS and stored at 4°C in PBS with 100 units/ml penicillin, 100 µg/ml streptomycin and 250 ng/ml Amphotericin B (Sigma-Aldrich) for up to 3 weeks before use in cell culture.

### Generation of rat precision cut lung slices and culture in the stretch device

Precision-cut lung slices (PCLS) were prepared from 19–21 weeks old male Sprague Dawley rats (540–680 g; Janvier, France) living under controlled conditions (22°C, 55% humidity, 12-h day/night rhythm and food and water *ad libitum*). Animal experiments were approved by the regional animal experiment ethical review board in Malmö/Lund (ethical no.: Dnr 5.8.18-09470/2021). Rat PCLS were prepared as previously described (Larsson-Callerfelt et al., 2013). Rats were euthanized with an overdose of pentobarbital (intraperitoneal, 150 mg/kg). Then, the trachea was fixed with a tracheal cannula and the lungs were filled with 25 ml of pre-warmed low melting agarose (Sigma-Aldrich) solution (1%) through the trachea and then chilled with ice. The thorax was then opened and the heart–lung package was removed and put on ice to further solidify the agarose. The lung lobes were cut into 5–10 mm thick tissue cylinders with a coring tool generating parenchymal cores with a diameter of 10 mm without any larger airways and vessels. The cores were cut into 350 μm thick slices using a Krumdieck tissue slicer (Alabama Research and Development, Munford, AL, United States) that was filled with ice cold DMEM/F12 with 1% (v/v) antibiotic and antimycotic solution (AB/AM, Sigma-Aldrich). Slices were collected and transferred to a petri dish with 25 ml of complete culture medium, namely DMEM/F12 with 1% (v/v) AB/AM and 1% (v/v) Insulin-Transferrin-Selenium (ITS -G, Gibco, Thermo Fisher Scientific), and placed in a humidified incubator (37°C and 5% CO2). The medium was changed every half hour for the first 2 hours and then every hour for the next 2 hours to remove agarose and release of generated inflammatory mediators from the airways and blood vessels. Slices were incubated in medium overnight before mounting in the stretch device, ∼24 h after euthanasia of the animal. The PCLS were mounted on top of the elastic silicon membrane in the wells of the stretch device and all the other components were assembled as described above. Then, half of the PCLS were cultured under cyclic stretch (CS; 15% theoretical stretch from a 5% baseline with a frequency of 16 cycles per minute, same as for repopulated DLS, see below for details) or as non-stretched controls (ST, static) to compare the effect of the stretch on the slices. In every case, 1 ml of complete culture medium was added for culture at 37°C and 5% CO_2_ in a humidified incubator.

### Experimental characterization of the achieved strain in DLS and PCLS

Tissue slices (DLS or PCLS) were mounted in the wells of the stretch device as described above. The stretch movement was calibrated with different theoretical values, namely 0.2, 5, 10 and 15%, in a pseudocyclic manner (increasing and decreasing the theoretical stretch) for a total of 3 cycles. The theoretical values were set in the Arduino programme to each specific stretch value and left to stabilize for at least 60 s after the piston reached and stopped at the aimed value. Two DLS and two PCLS were measured independently in a single well of one stretch device. The images were obtained with a stereomicroscope (Olympus SZ-STU1 and SZ-STB1 arms, and SZ40 objective, Olympus, Japan). A customized 3D-printed device was used for coupling a camera from a mobile telephone to the binoculars of the microscope for image acquisition. Every image was independently calibrated considering the size of the hole in the well (7 mm).

In addition, the stretch movement was evaluated in each well of each stretch device (8 wells per device, for a total of 16 wells) to assess reproducibility. The same theoretical stretch used for the repopulated DLS and PCLS (5%–15%, or 10% amplitude) was used in this case. The images to quantify the stretch movement were obtained as described above for the calibration measurements, setting the theoretical stretch to either 5 or 15% stretch and letting it to stabilize at each value. One DLS was used for the eight wells of each stretch device (two DLS in total).

In every case, the stretch movement was measured by drawing lines between specific points in the lung slices (e.g., alveolar wall intersections) that could be tracked along all the images from each set, as shown in Supplementary Figure S1. The lines were drawn in the central parts of the wells overlying the piston where radial and circumferential strain occur in a horizontal plane. Towards the edges, the tissue strain also occurs vertically and is not possible to reliably quantify it from birds-eye perspective. These lines were used as diameters of circles which area was used for the comparisons. Three lines (i.e., circle areas; the same for each set of images) were drawn in each image and the average change in circle area was calculated from the three sets of measurements.

### Cell culture

NCI-H441 epithelial cells (ATCC, HTB-174) were subcultured in T75 flasks according to instructions provided by ATCC. Specifically, cells were cultured in RPMI 1640 medium including GlutaMax™, phenol red and HEPES (Thermo Fisher Scientific, MA, United States) supplemented with 10% (v/v) HyClone Fetal Clone III serum (Cytiva, MA, United States), 1 mM sodium pyruvate (Sigma-Aldrich) and 1% (v/v) AB/AM. Cells were cultured at 37°C and 5% CO_2_ in a humidified incubator and used in passage 55 to 64. When confluent, cells were detached using TrypLE Express Enzyme (Gibco, Thermo Fisher Scientific) after removing the medium and rinsing with Dulbecco’s Phosphate Buffered Saline (DPBS; Sigma-Aldrich), centrifuged for counting and then resuspended in a known volume of complete medium for subculturing (1:3 to 1:8 ratio) or for seeding on the DLS.

### Cell seeding and culture in DLS mounted in the stretching device

Prior to cell seeding, 500 µL of complete medium were added onto the mounted DLS and incubated at 37°C and 5% CO_2_ during 16–24 h for equilibration. Subsequently, the pre-equilibration medium was removed, and 500,000 cells were seeded on top of each DLS in 50 µL of complete medium (enough to cover the surface of the DLS exposed to the well), left to attach for 2 h at 37°C and 5% CO2, and then 950 µl of complete medium were added to the well for culture during 1 or 4 days. These repopulated DLS are simply termed “lung slices” hereinafter. The same mounting and seeding procedure were followed in every experiment for samples subjected to CS and for ST controls. The stretch device was started for stretching (where applicable) after the attachment of cells and addition of the medium up to 1 ml. A setting of 16 cycles per minute with a theoretical amplitude of 10% increase in surface area from a baseline 5% stretch was used throughout the experiments, with the change in stretch following a sinusoidal curve (Figure 1D). The frequency of 16 cycles per minute was selected considering that the respiratory rate in a healthy human adult at rest is between 12 and 20 breaths per minute, being 16 in the middle of this range (Rolfe, 2019; Nicolò et al., 2020). The same value was used to evaluate the effect of the stretch in rat PCLS integrity and cell viability even if rats have a higher breathing rate, since the final aim is to use human PCLS as a model. As a regular cell culture control, 250,000 cells were seeded in 12-well tissue culture plastic (TCP) in 1 ml of complete medium.

### Metabolic activity measurement

A 10% solution of the resazurin-based PrestoBlue HS Cell Viability Reagent (Thermo Fisher Scientific) was prepared in complete medium and 500 µl of this solution were added to PCLS or H441 cells for measurement of their metabolic activity in the different conditions (ST, CS and TCP). The cultures were incubated at 37°C, 5% CO_2_ for 3 and 2 h for the PCLS and H441 cells, respectively, in static conditions, i.e., without stretching. Afterwards, the diluted reagent was retrieved and resazurin reduction was measured by spectrophotometry (absorbance) at 570 and 600 nm. The percentage of reduction for each sample was calculated using the following equation from the manufacturer’s instructions:

$$\frac{O_{600}\times A_{570}-O_{570}\times A_{600}}{R_{570}\times N_{600}-R_{600}\times N_{570}}\times 100$$

where *O*_570_, *O*_600_, *R*_570_ and *R*_600_ are the molar extinction coefficients for the oxidized (*O*) and reduced (*R*) PrestoBlue reagent at 570 and 600 nm. *N*_570_ and *N*_600_ are the absorbance values of the blank wells (PrestoBlue reagent incubated in the absence of cells) at 570 and 600 nm, respectively, while *A*_570_ and *A*_600_ are the absorbance values of the unknown samples. The actual values used were calculated in previous experiments and are as follows: *O*_570_ = 80,586, *O*_600_ = 117,216, *R*_570_ = 155,677 and *R*_600_ = 14,652.

### RNA isolation

Samples from the different groups of the lung slices culture were retrieved after 1 and 4 days for RNA isolation. Briefly, samples were rinsed with warm DPBS and 100 µL of RNAlater (Sigma-Aldrich) were added to the wells for RNA stabilization. Then, the well was disassembled, and the lung slice was carefully collected and placed in a tube containing 100 µL of RNAlater. In the case of the TCP samples, the cells were detached from the surface of the well with a cell scraper after the addition of the RNAlater solution. The samples were kept for 1 h at room temperature and then transferred to −80°C until further processing.

The RNA isolation was performed using the Qiagen RNeasy Mini kit (Qiagen, Germany). First, the samples were thawed at room temperature and five times the RNAlater volume of PBS (500 µl) was added to before centrifugation to aid the removal of RNAlater. Afterwards, the lung slice samples were resuspended in 600 µl of the lysis buffer included in the kit and disrupted and homogenized with a sterile Hard Tissue Omni Tip (OMNI International, GA, United States). Then, samples were centrifuged for 3 min at 16,000 g and the supernatant was transferred to a new tube for the actual RNA isolation following the manufacturer’s standard protocol. The concentration of the eluted RNA was measured with a NanoDrop 2000c instrument (Thermo Fisher Scientific) and the samples were stored at −80°C until further processing.

### Quantitative PCR

First, the samples were thawed at room temperature and a volume corresponding to 600 ng of RNA from each sample was transferred to a PCR tube for retro-transcription. Subsequently, the cDNA was synthesized with the use of the QuantiTect Reverse Transcription kit (Qiagen) following the manufacturer’s instructions, including an initial genomic DNA elimination step. cDNA samples were stored at −20°C until further use.

The quantitative PCR (qPCR) was performed with the Quantifast SYBR Green Master Mix (Qiagen). 20 ng of cDNA template from each sample were used per reaction, which were added into Multiplate 96-well PCR Plates that were sealed with the Microseal ‘B’ Film PCR Plate Heat Seal (both from Bio-Rad, CA, United States). The measurements were performed in a Mx3005P Real-Time PCR System (Agilent Technologies, CA, United States) after centrifugation at 2000 *g* for 2 min. The thermal profile included an initial heat activation for 5 min at 95°C, followed by 40 cycles of a denaturation step of 10 s at 95°C and a combined annealing/extension step for 30 s at 60°C, performing the fluorescence measurement right after it. Dissociation curves were obtained for every run to confirm the correct hybridization of the primers. All the primers used were obtained as validated QuantiTect Primer Assay probes (Qiagen) for the detection of collagen III α1 (COL3A1), collagen IV α3 (COL4A3), laminin α5 (LAMA5) and SFTPB transcripts in SYBR green-based qPCR reactions (see Supplementary Table S2 for information on the primers). Data obtained from the qPCR runs was curated in R (version 4.1.1) for the calculation of ΔΔC_T_ values and subsequent logarithmic values (2^−ΔΔCT^) for the representation of relative expression as fold change of the control group (TCP at day 1) (Livak and Schmittgen, 2001). Only C_T_ values below 35 cycles were used for the analysis, considering that non-specific primer binding could be found above this value. In addition, peptidylprolyl isomerase A (PPIA) was used as reference/housekeeping gene for the calculation of ΔC_T_ values. The geometric mean of the biological replicates of the control group (TCP at day 1) was used for the estimation of the ΔΔC_T_ for each sample in order to decrease the influence of extreme data points (Vandesompele et al., 2002). Relative expression values were graphed and analyzed in GraphPad Prism (version 9.2.0).

### Histology and immunohistochemistry

#### Sample retrieval

After 1 and 4 days for the lung slices and 1 and 3 days for the PCLS, samples from each group were collected for histological and IHC processing and analysis. Specifically, the medium was aspirated and pre-warmed DPBS was added to the wells for washing, followed by 1 ml of 4% formaldehyde for fixation during 1 h at room temperature. Finally, the formaldehyde solution was removed, and samples were washed with PBS. This solution was used to keep the samples hydrated during storage at 4°C for up to 24 h until further processing.

#### Sample dehydration and paraffinization

For dehydration, the PBS was removed from the wells, these were disassembled, and the samples were carefully collected and placed in a 70% ethanol bath. Then, for the lung slices, the central part (the one in contact with the medium in the well) was separated with a Ø8 mm biopsy punch, introduced in a nylon tissue bag and then put into a histology cassette. The same procedure was used for PCLS, except for the punching, which was not needed since these slices were prepared to fit the size of the well in the stretch device. Each lung slice/PCLS was treated individually in different cassettes. The dehydration involved the following steps: 1 h in 70% ethanol, 1 h in 95% ethanol, 30 min in 99.5% ethanol, 15 min in a 1:1 mixture of 99.5% ethanol and xylene (VWR, United States) and finally 30 min in xylene. Then, samples were introduced in a first paraffin bath for 1 h and after that in a second paraffin bath for 30 min. Subsequently, samples were embedded in paraffin and blocks were stored at room temperature until sectioning.

#### Sample sectioning

Sections of 4 µm thickness were obtained with a HM 355S Automatic Microtome (Thermo Fisher Scientific) with circulating water system and coupled to a water bath set at 39°C for optimal section flattening. The sections were collected on SuperFrost or SuperFrost Plus Menzel Gläser slides (VWR, PA, United States) and left to dry for 16–24 h.

#### Hematoxylin and eosin staining

Hematoxylin and eosin (H&E) staining of cells within lung slice/PCLS sections was performed following a well-established protocol. Briefly, the slides to stain were first placed in an oven at 56°C for 30 min to ensure adherence of the sections to the slides. Then, they were deparaffinized and rehydrated in xylene for 5 min (two times), 99.5% ethanol for 5 min (two times), 95% ethanol for 5 min, 70% ethanol for 2 min and distilled water for 2 min. Then, the sections were introduced in a solution of Mayer’s Hematoxylin (Histolab, Sweden) for 6 min, followed by a bluing for 6 min in running tap water. Subsequently, the sections were immersed in eosin (Histolab) for 30 s and subsequently left under running tap water for 30 s. Finally, the samples were dehydrated again in 95% ethanol for 30 s, 99.5% ethanol for 1 min (two times) and xylene for 2 min (two times), mounted with the hydrophobic mounting medium Pertex (Histolab) for sealing with a coverslip and stored at room temperature. Brightfield images were obtained in an Olympus VS-120 slide scanner (Olympus, Japan) and processed in QuPath (version 0.3.0) and ImageJ (version 1.53f51).

#### Immunohistochemistry

The slides intended for IHC were initially placed in the oven at 56°C for complete attachment of the paraffin sections to the slide surface. Then, they were deparaffinized and rehydrated in xylene for 5 min (two times), 99.5% ethanol for 4 min (two times), 95% ethanol for 4 min, 70% ethanol for 4 min and a short dip in ultra-pure (milliQ) water. Then, the slides were placed in the holders for the PT Link instrument (Agilent Technologies) used for antigen retrieval, which was filled with a pH 6 (citrate-based) or pH 9 (Tris/EDTA-based) EnVision FLEX Target Retrieval Solution (Agilent Technologies) for heat-induced epitope retrieval (HIER) of sections intended for Ki67 or YAP staining, respectively, during 30 min at 97°C. Afterwards, the slides were washed in PBS (5 min, three times) and the sections were encircled with a hydrophobic pen. Subsequently, 50 µl of the primary antibody dilution (diluted in 2% (w/v) BSA in PBS) was added to each lung slice section and they were incubated for 1 h at room temperature in a humidified chamber. Two different double immunostainings were performed, one including an anti-active Yes-associated protein 1 (YAP1) antibody (Abcam, UK; 1:500) plus an anti-pan-cytokeratin (pan-CK) antibody (Abcam, UK; 1:100), and the other an anti-Ki-67 antibody (Abcam, UK; 1:500) mixed with the same anti-pan-CK antibody (see Supplementary Table S3 for more information about the antibodies used in this work). After the primary antibody incubation, the slides were washed in PBS (5 min, three times) and 50 µL of the secondary antibody solution, together with a DAPI ready-made solution, (Sigma-Aldrich), were added onto the sections. This solution included a goat anti-rabbit IgG conjugated to Alexa Fluor Plus 647 (Thermo Fisher Scientific, MA, United States; 1:200) targeting either the anti-active YAP1 or the anti-Ki-67 antibody, and a donkey anti-mouse IgG conjugated to Alexa Fluor 555 (Thermo Fisher Scientific, MA, United States; 1:200) targeting the anti-pan-CK antibody (Supplementary Table S3). They were incubated for 45 min at room temperature and subsequently mounted with the Dako Fluorescent Mounting medium (Agilent Technologies) and sealed with a coverslip. They were kept at room temperature for 16–24 h and subsequently stored at 4°C before and after imaging in an Olympus VS-120 slide scanner (Olympus, Japan). Images from four different channels were obtained, with the signal detected in the FITC one corresponding to the autofluorescence of the DLS fibers (mostly collagen and elastin), which was used to discard false positive cells detected in the analysis due to the autofluorescence in the DAPI channel. The other channels used were Cy3 and Cy5 for the observation of pan-CK and YAP1/Ki-67, respectively. The images were analyzed with the QuPath software (version 0.3.0) for positive cell detection, using the same parameters and thresholds for all the images from each staining (YAP1 and Ki-67) (Bankhead et al., 2017). Only the signal from the YAP1 or Ki-67 channel that co-localized with the nuclei was considered for detecting positive cells for those markers. The data obtained was represented and analyzed in GraphPad Prism (version 9.2.0).

### PCLS viability in the stretch device

PCLS were divided in three groups (*n* = 3 PCLS from the same rat per group): CS for those subjected to cyclic stretch, ST for those cultured under static conditions mounted in the well designed for the stretch device, and free floating (FF), i.e., kept in a 35 mm culture dish floating in medium as control. PCLS were mounted/cultured as described above and kept in culture for 24 h at 37°C, 5% CO_2_ in DMEM/F12 with 1% (v/v) AB/AM. In the case of CS culture, the PCLS were subjected to a 10% cyclic stretch (in amplitude, from a 5% baseline) with a frequency of 16 cycles per minute, as described above. Subsequently, the medium was removed, and the PCLS were washed in PBS and stained with a Live and Dead Cell Assay solution (Abcam, ab115347; 1:200 in PBS) supplemented with Calcein AM Viability Dye (eBioscience, Thermo Fisher Scientific) at a concentration of 2 µM. The PCLS were directly imaged in the well of the stretch device by pushing the glass towards the tissue and the silicon membrane with extra magnets after removing the outer and inner wells. A coverslip was placed on top of the glass of the well for imaging in an inverted Nikon Eclipse Ti2 fluorescence microscope (Nikon) controlled with the NIS Elements AR software (Nikon). Images covered the whole area of the part of the tissue exposed to the hole for the piston (for CS and ST) or the whole PCLS (FF), and were acquired as a Z-stack spanning the full thickness of the PCLS. Images were corrected for brightness and contrast and presented as a maximum intensity Z-projection.

### Statistical analysis

Data was represented and analyzed in GraphPad Prism (version 9.2.0). A two-way ANOVA was used for the stretch comparison, with cycle and slice type (DLS or PCLS) and specimen replicate (1 or 2) as one variable and theoretical stretch percentage as the other variable, and using a Tukey’s test with multiple comparisons correction as post-hoc analysis. A Kruskal-Wallis followed by Dunn’s multiple comparisons post-hoc test was performed for comparison between groups and timepoints in all the other cases. Results were reported as individual data points with group median, accepting as statistically significant a *p*-value < 0.05. (**) *p* < 0.01 and (*) *p* < 0.05. Data is mentioned in the text as mean ± SD. Five biological replicates were used for lung slices (*n* = 5, unless otherwise stated), while three biological replicates were used in the experiments involving PCLS (n = 3, unless otherwise stated). Each lung donor/rat is considered a biological replicate for the lung slices/PCLS (both in CS and ST conditions) and each experiment/plate for TCP.

## Results

### Experimental characterization of the achieved strain in DLS and PCLS

Two DLS and two PCLS were used to calculate the stretch induced at a specific theoretical stretch value. As shown in Supplementary Figure S2, there were slight differences between PCLS and the DLS, with no significant variations between cycles. Furthermore, the experimental stretch values were found to be higher than the theoretical ones overall, with a lower difference in the case of the PCLS (8.6% experimental stretch vs. 5% theoretical stretch, 14.1% vs. 10% and 18.1% vs. 15%) than for the DLS (12.4% vs. 5%, 19.4% vs. 10% and 26.1% vs. 15%), where the difference was more noticeable. However, we only found statistical differences when comparing PCLS 1 cycle 3 with DLS 1 cycle 1 (*p* = 0.03), and PCLS 1 cycle 3 with DLS 1 cycle 3 (*p* = 0.03). No detachment from the membrane or folding of the DLS/PCLS was observed for the samples analyzed in the whole study. A movie of the movement of a mounted DLS is included in Supplementary Video S1.

In another set of experiments, we characterized the reproducibility of the stretch in the different wells of the two stretch device units available, using one DLS per unit. As observed in Supplementary Figure S3, we found non-significant slight differences between the wells within the same device unit, but not between units.

### Cell culture of H441 epithelial cells on DLS under cyclic stretch and static conditions

**FIGURE 2:**
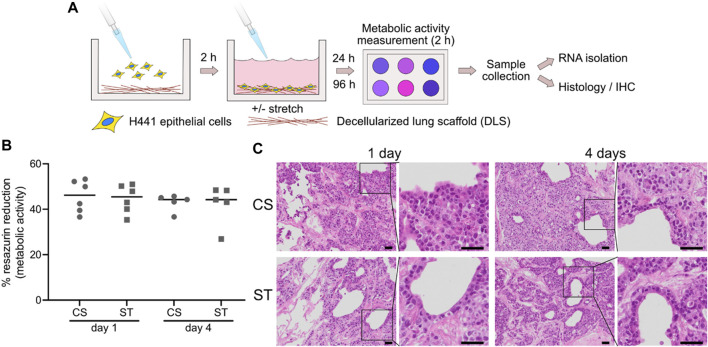
Experimental overview and lung slice repopulation. H441 cells were seeded into wells with mounted DLS and exposed to mechanical stimuli for 1 or 4 days, after which samples were collected **(A)**. Cell metabolism was evaluated after 1 and 4 days of culture by a resazurin-based reduction assay **(B)**. CS: cyclic stretch (circles), ST: static culture (squares). Data points represent data from individual lung slice samples (*n* = 6 for both conditions at day 1, and *n* = 5 at day 4), horizontal lines show group median. Representative images of hematoxylin and eosin (H,E) stained repopulated lung slices after 1 and 4 days of culture, with (CS) or without (ST) cyclic stretch **(C)**. Scale bars are 50 µm.

Epithelial H441 cells were cultured in human DLS under cyclic stretch (CS) and static (ST) conditions. A schematic representation of the experimental procedure can be found in Figure 2A. DLS were produced and cell nuclei could not be observed, showing successful decellularization, in agreement with previously published studies using the same decellularization protocol (Rosmark et al., 2018; Elowsson Rendin et al., 2019) (Supplementary Figure S4). As a first step, the cells were successfully seeded into the DLS to give repopulated lung slices and left in culture for 24 and 96 h (1 and 4 days). At these timepoints, the cell viability was evaluated by a resazurin-based assay. No significant differences were seen between the cell culture conditions (CS and ST) or timepoints (1 and 4 days), with similar percentage of reduction values (Figure 2B). Specifically, the reduction for CS was 45.69 ± 7.06% at 1 day and 43.00 ± 3.67 at day 4, compared to 44.61 ± 6.19 and 42.21 ± 8.88 for the ST condition. Lung slices were retrieved after the metabolism measurements and processed for either qPCR analysis or histology and IF. The histological analysis by H&E staining showed that the H441 cells attached to the DLS structure and proliferated, filling the spaces found in the scaffolds, not growing merely as monolayers (Figure 2C). The magnified areas in Figure 2C show areas where open lumens are encircled by cells with an appearance resembling cuboidal bronchiolar epithelium.

### Assessment of the proliferative state of H441 cells in CS and ST culture conditions

**FIGURE 3:**
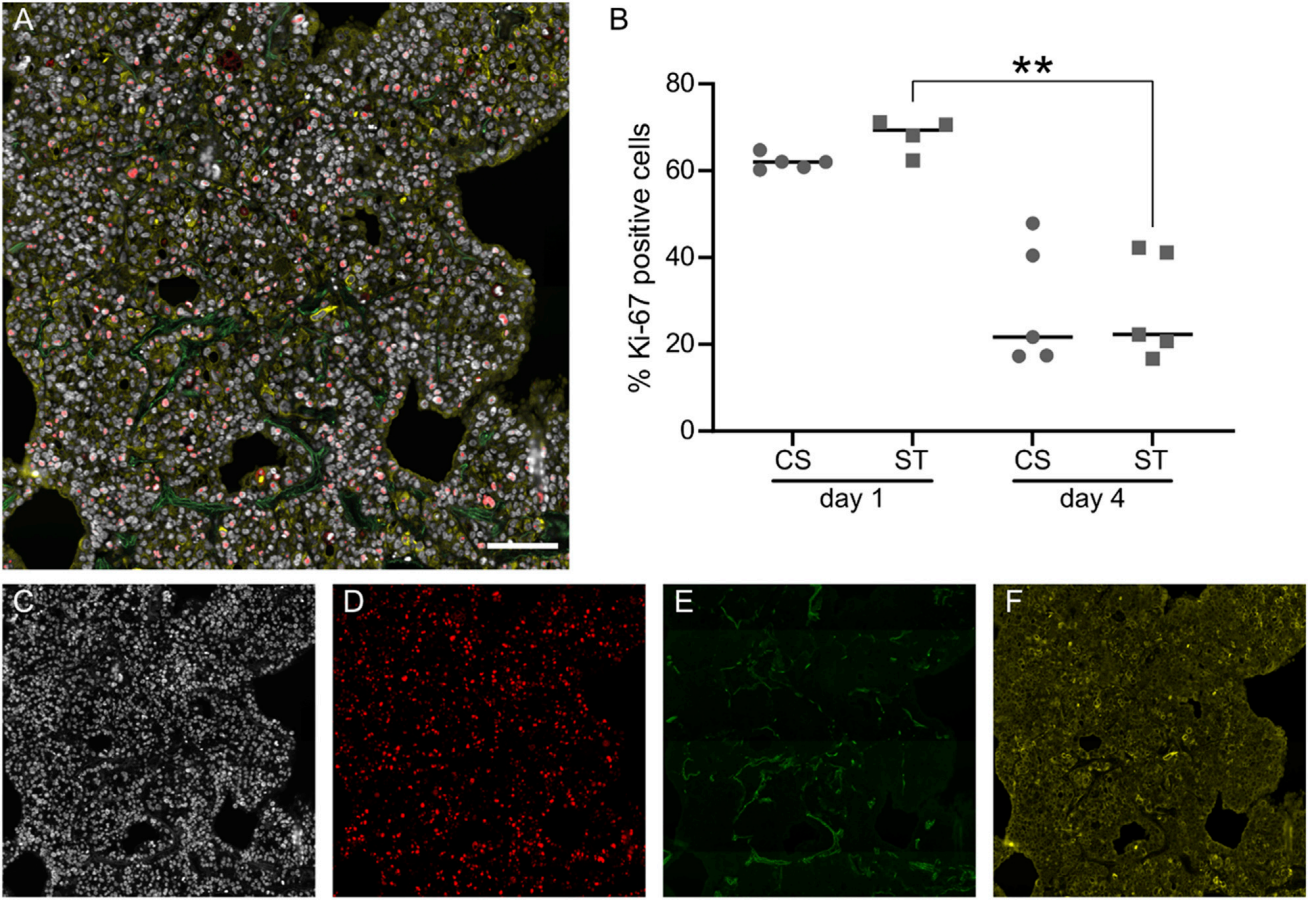
Evaluation of cell proliferation with Ki-67 staining and quantification. Representative image of lung slices stained against Ki-67 (red), epithelial marker pan-cytokeratin (yellow) and nuclei (DAPI, white), while autofluorescence at 495/519 nm (FITC) was used to visualize ECM fibers in the lung slice **(A)**. Quantification of Ki-67 in cell nuclei expressed as proportion of positive cell nuclei **(B)**, data points represent data from individual lung slice samples, horizontal lines show group median. Individual channels from **(A)** are shown in **(C–F)**. Scale bar is 100 μm, CS: cyclic stretch (circles), ST: static culture (squares), **: *p*-value < 0.01.

For the evaluation of the proliferative state of the epithelial cells, we immunostained for Ki-67, a widely used proliferation marker (Sun and Kaufman, 2018). As observed in Figure 3A, the signal for Ki-67 co-localized with the nuclei (DAPI), allowing the quantification of the proportion of proliferative cells in the whole slide imaging software QuPath (Figure 3B). The number of Ki-67 positive/proliferative cells for the CS culture decreased from (62.01 ± 1.73) day 1 to (28.96 ± 14.24) day 4, although not statistically significant. The number of proliferative cells showed a similar decreasing trend for the ST group from (68.09 ± 4.03) day 1 to (28.62 ± 12.13) day 4. Figures 3C–F shows the individual channels from the composite image (Figure 3A), which were used for the calculation of the positive cell number percentages. More images can be observed in Supplementary Figure S5.

### Evaluation of YAP1 activation in CS and ST culture conditions

**FIGURE 4:**
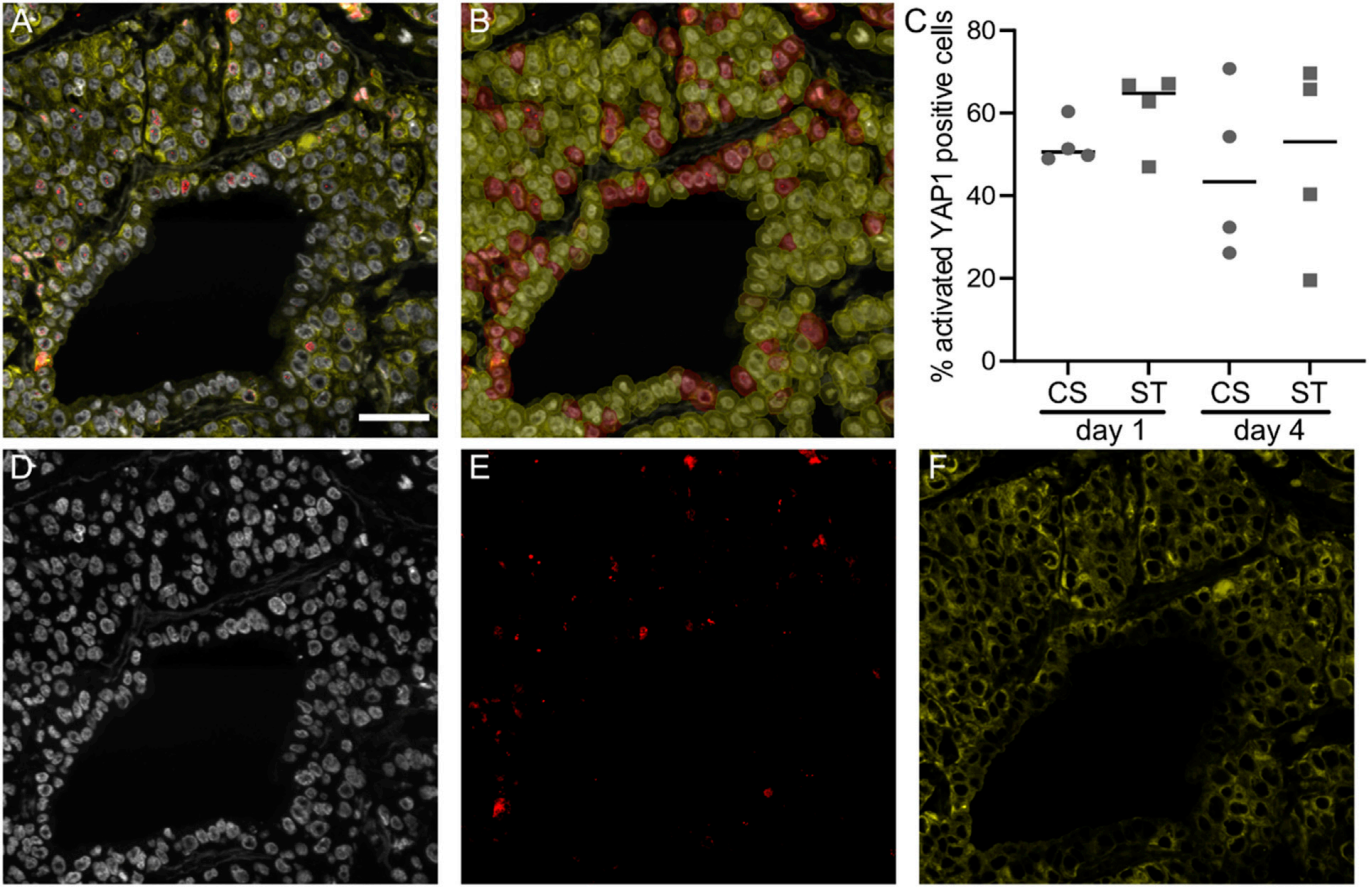
Activated YAP1 staining and quantification. Representative image of lung slices stained against activated YAP1 (red), epithelial marker pan-cytokeratin (yellow) and nuclei (DAPI, white), **(A)**. Classification overlay from the QuPath software showing cell classification from the area shown in **(A,B)**, activated YAP1 staining was thresholded in cell nuclei to classify cells as either positive (magenta) or negative (yellow). Quantification of activated YAP1 positive cells **(C)**, data points represent data from individual lung slice samples, horizontal lines show group median. Individual channels from **(A)** are shown in **(D–F)**. Scale bar is 50 μm, CS: cyclic stretch (circles), ST: static culture (squares).

We investigated the activation of YAP1 in the different culture conditions (CS and ST) by immunostaining with an antibody specific for the active (non-phosphorylated) form of the protein, in combination with the pan-CK epithelial cell marker and nuclei (DAPI) counterstaining, as shown in Figure 4A. YAP1 is a downstream transcriptional factor in the Hippo signaling pathway, known to be involved in mechanotransduction for the regulation of embryogenesis and epithelial homeostasis (Lange et al., 2015). This arrangement allowed us to efficiently segment cells in the QuPath software, and only consider as YAP1 positive cells a YAP1 signal localized to the nucleus (Figure 4B). This quantification (Figure 4C) did not show an obvious stretch-induced effect on activated YAP1. The percentage of positive cells day 1 was 60.91 ± 9.51 for the ST and 52.62 ± 5.24 compared to CS, with similar values day 4, 48.83 ± 23.44 for ST and 45.92 ± 20.50 for CS. Images in Figures 4D–F show the individual channels in Figure 4A, used for the calculation of the YAP1 positive cell percentages.

### Expression analysis of ECM and surfactant protein B genes in H441 cultures

To study the changes in the expression of ECM and surfactant coding genes induced by cyclic stretch, we isolated RNA for qPCR analysis from lung slices at 1 and 4 days. We also used TCP-cultured cells as a control representing common culture conditions, which could enable us to decode the differences induced by the 3D microenvironment that the lung slices provide to the cells. The relative expression levels for all groups and timepoints are expressed as fold change (mean ± SD, where actual values are mentioned) in comparison with the TCP group at day 1. With these settings, we evaluated the relative expression of COL3A1, COL4A3 and LAMA5 as ECM proteins relevant in distal lung epithelium, and SFTPB, which has been shown to be expressed in H441 cells (Rucka et al., 2013).

**FIGURE 5:**
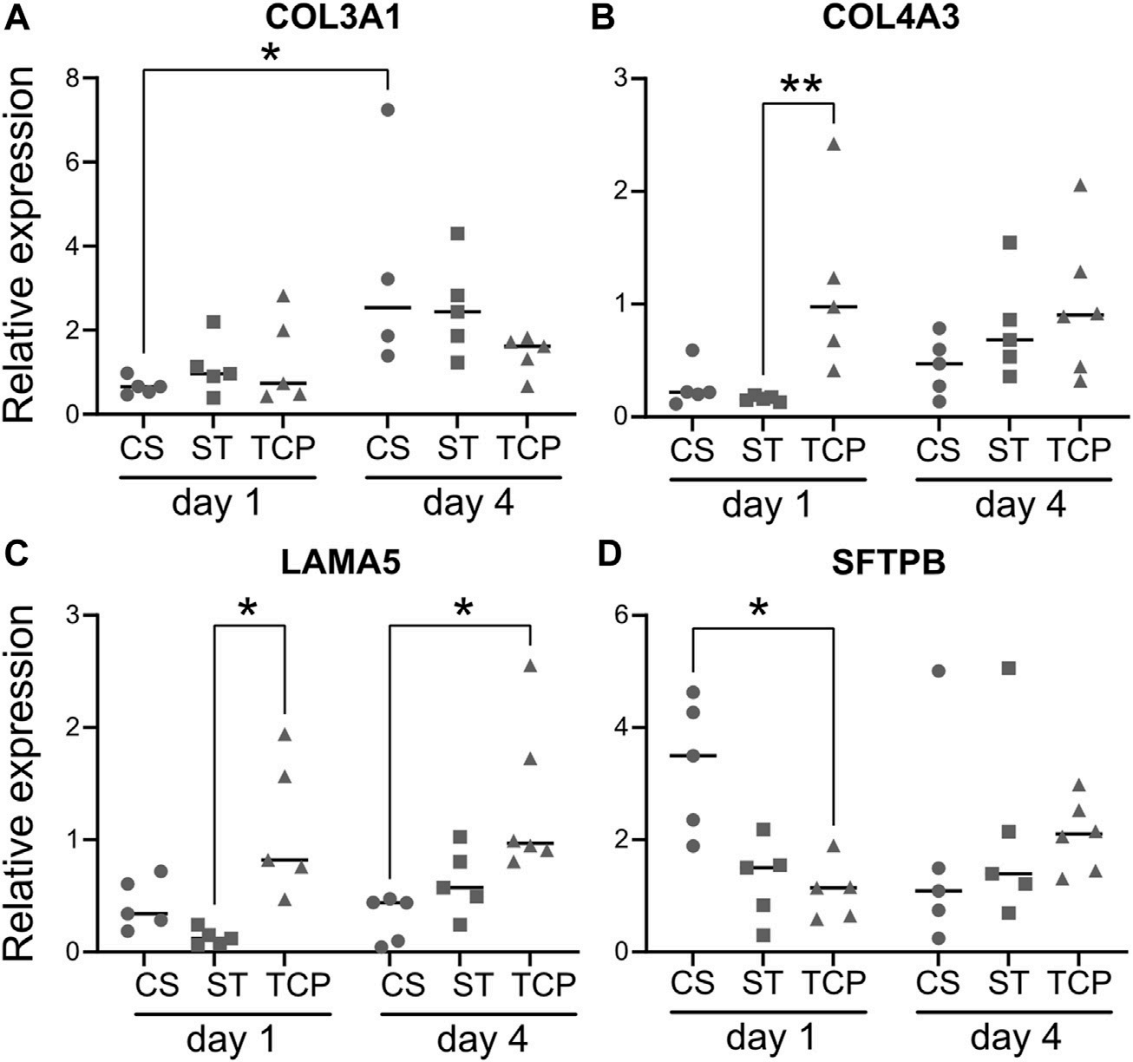
Quantitative PCR analysis of the expression of ECM and surfactant genes. Gene expression results for collagen type III α1 chain (COL3A1, **(A)**, collagen type IV α3 chain (COL4A3, **(B)**, laminin α5 (LAMA5, **(C)** and surfactant protein B (SFTPB, **(D)** for the H441 epithelial cells cultured in the different conditions tested. Data is presented as relative expression compared to TCP at day 1, with individual points (biological replicates or “n”) together with the median. CS: cyclic stretch (circles), ST: static culture (squares), TCP: tissue culture plastic (triangles), *: *p* < 0.05. *n* = 5 for every gene for CS and ST, except for CS at day 4 for COL3A1 (*n* = 4). *n* = 3 for TCP in every case.

In the case of COL3A1 (Figure 5A), there is a trend of increasing expression from day 1 to day 4 for each group, more pronounced in repopulated DLS than for TCP cultures, with a statistically significant difference for the repopulated DLS with CS (from 0.66 ± 0.20 to 3.43 ± 2.66 for CS, and 1.12 ± 0.67 to 2.53 ± 1.16 for ST). Comparing repopulated DLS with TCP culture, we observed higher COL4A3 expression (Figure 5B) for the TCP condition (1.12 ± 0.65) compared to DLS (CS: 0.27 ± 0.19, ST: 0.16 ± 0.02), with a significant difference found between ST and TCP (*p* < 0.05). Moreover, gene expression increased for the DLS from day 1 to day 4 (CS: 0.45 ± 0.26 and ST: 0.80 ± 0.46 at day 4; not statically significant), while the expression in cells cultured on TCP remained comparable (0.99 ± 0.65 at day 4). LAMA5 expression levels followed a very similar trend, with DLS showing lower values than TCP at day 1 (*p* < 0.05 for the comparison between ST and TCP) and increasing towards 4 days of culture (Figure 5C).

In contrast to the ECM-related genes, the expression levels of SFTPB in the cells cultured in repopulated DLS under CS conditions (3.33 ± 1.19) were increased in comparison to ST (1.27 ± 0.73) and TCP (1.22 ± 0.65) at day 1, although the differences were not statistically significant with stringent non-parametric test (Figure 5D). At day 4, the relative expression values showed a decrease for the CS samples (1.71 ± 1.90), whereas they were higher for the ST (2.10 ± 1.73) and TCP (2.08 ± 0.61) groups, in comparison with the day 1 values (no statistically significant differences).

### PCLS culture and viability under cyclic stretch

**FIGURE 6:**
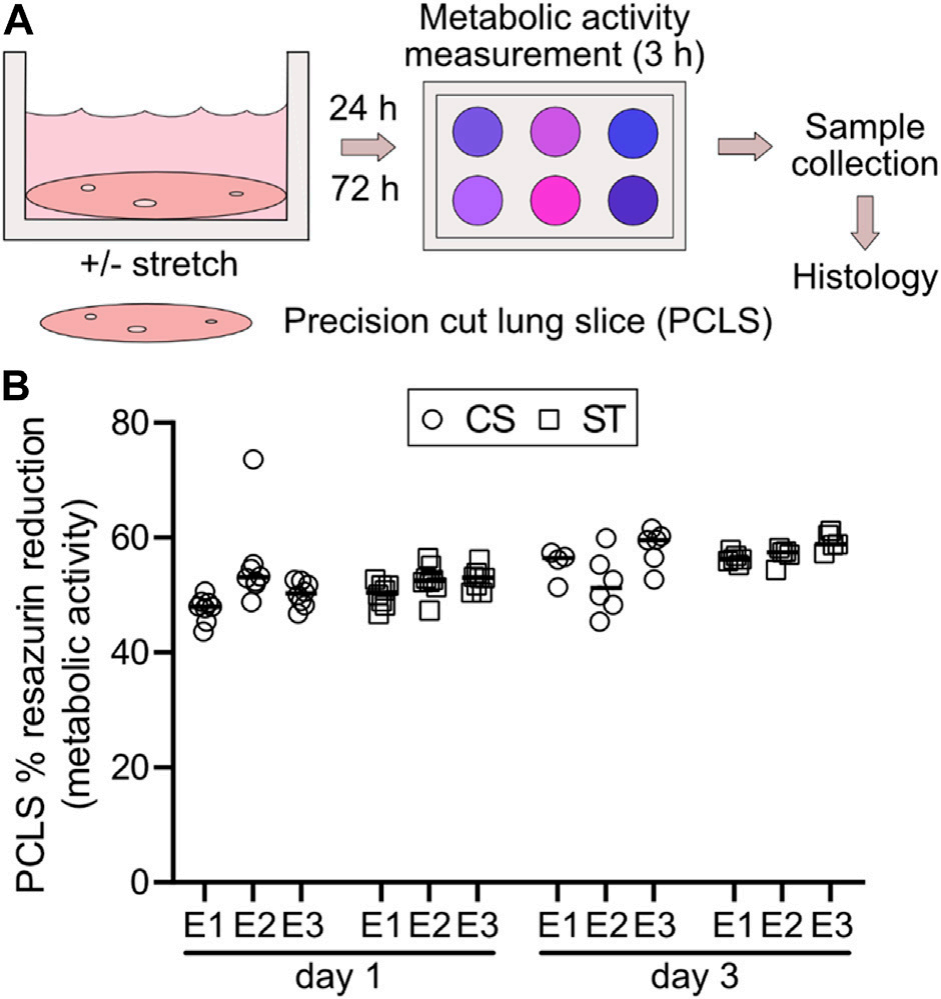
Experimental overview of PCLS culture and metabolic activity measurement with or without cyclic stretch. Rat PCLS were mounted in the stretching device and exposed to a physiomimetic mechanical stimulus for evaluation of PCLS viability in terms of metabolic activity and qualitative histological analysis after 24 h (1 day) and 72 h (3 days), in comparison to the static control counterpart **(A)**. Cell metabolism was assessed after 1 and 3 days of culture by a resazurin-based assay **(B)**. Data points represent individual PCLS samples within each experiment (E1-3), horizontal lines show group median for each experiment. CS: cyclic stretch (circles), ST: static culture (squares). *n* = 3 experiments with 8 and 4-6 technical replicates for both conditions at day 1 and day 3, respectively.

Rat PCLS were mounted on top of the elastic silicone membranes and cultured under cyclic stretch (CS) and static (ST) conditions (Figure 6A). In this case, the culture contained the various cell types found in the distal lung niche. The PCLS were placed in culture in the wells of the stretch device 24 h after euthanasia of the animals and cultured there for an additional 24 or 72 h (1 or 3 days). As observed in Figure 6B, no significant differences in viability were found between the culture conditions (CS and ST) or timepoints (1 and 3 days), with very similar percentage of resazurin reduction values in every case (Figure 6B). The mean reduction for CS at 1 day was 51.00 ± 3.92%, CS at 3 days was 54.93 ± 4.12%, ST at 1 day was 51.74 ± 1.54%, and ST at 3 days was 56.84 ± 1.84%. The PCLS were then retrieved after 3 days and processed for histology.

**FIGURE 7:**
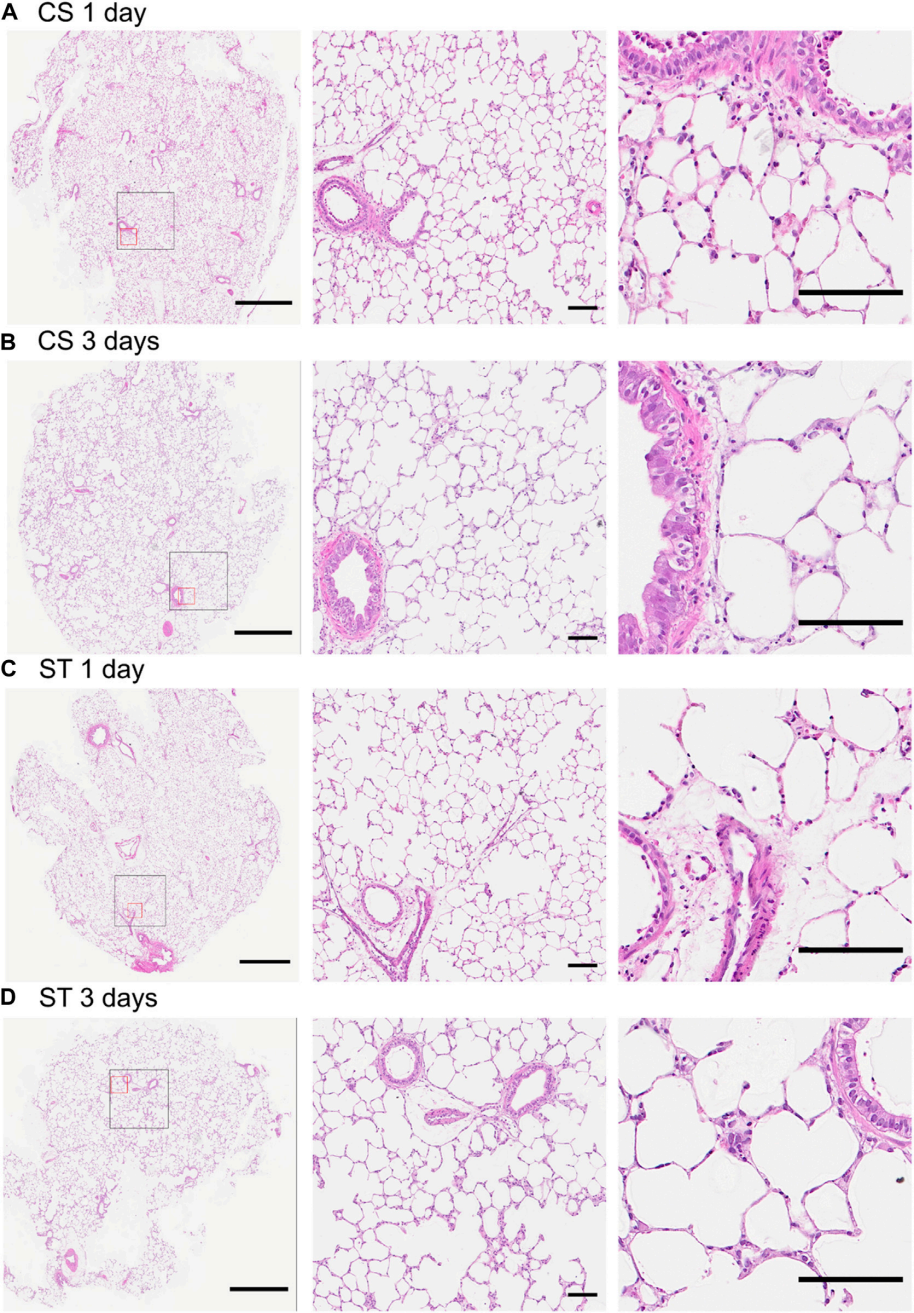
Hematoxylin and eosin staining of PCLS cultured under cyclic stretch or static conditions. Representative images of stained sections exposed to cyclic stretch (CS) for 1 **(A)** and 3 days **(B)** and static controls (ST) at these same timepoints (**(C,D)**, respectively), including overview (left) and higher magnification (center and right) images. The black and red squares in the overview image are displayed in higher magnification to the right of the overview. Samples were retrieved after 1 and 3 days of stretch (2 and 4 days after slicing). Stained sections from two technical replicates from each experiment (*n* = 3) and timepoint were evaluated. No difference between the timepoints nor between CS and ST groups were seen in morphological assessment focusing on cell shrinkage and pyknotic bodies as signs of apoptosis. Scale bar is 1 mm for the overview images (left) and 100 µm for the higher magnification ones (center and right).

Histological analysis by hematoxylin and eosin staining (Figure 7) showed that the PCLS cultured under cyclic stretch did not display evident morphological differences in comparison with the ones cultured under static conditions at each timepoint (1 and 3 days). Moreover, we could not observe any signs of cell shrinkage or pyknotic bodies, as indications of cell apoptosis, confirming that the stretch exerted on the PCLS did not affect their viability.

LIVE/DEAD staining of PCLS mounted in the wells (CS and ST, Supplementary Figure S6) did not show big differences in cell viability compared to the slices cultured in a tissue culture dish (FF) after 24 h. A qualitative assessment showed increased cell death mainly associated to larger airways and blood vessels in both mounted and FF slices, suggesting that this is inherent to the methodology used to obtain PCLS rather than the use of the stretch device. Mounted slices (CS and ST) also showed a higher number of dead cells in the edges when compared to FF, likely related to the dead cells in the part of the tissue pressed together just outside the well where the slices are held in place. Interestingly, no differences were observed between CS and ST in this regard, meaning that the stretch movement did not affect cell viability negatively and thus should not introduce differences between the CS and ST groups for the other readouts in this study. Overall, most of the cells in the PCLS appeared alive in every case, independently of stretch or culture condition.

## Discussion

In this work, we present a novel device aimed at mimicking the physiological conditions exerted during breathing, allowing for *in vitro* studies of lung cells cultured in a 3D microenvironment. The stretch movement in the device was characterized within the area of the piston (6 mm), which covers most of the area of the hole (7 mm) that exposes the tissue slice to the piston, and where a homogeneous strain is expected due to the trapezoid shape of the piston. The results show differences between the DLS and the PCLS, non-significant in most of the comparisons, which suggests an effect of the cells on the propagation of the stretch in the slice. It will be interesting to study this effect further, also with repopulated DLS. The higher experimental stretch values can at least in part be explained by the fact that the measurements for methodological reasons were performed in the central parts of the tissue. In this area, the average strain of the tissue slices is most likely lower, as the possibility for circumferential strain diminishes towards the slice edges where the movement is restricted by the well that keeps the slice in place. Nevertheless, it should be considered that at a 0% theoretical stretch the piston may not be in direct contact with the membrane, affecting the correspondence between the theoretical and the experimental values. Moreover, the slight non-significant differences found between the wells in the different stretch devices were assumed as normal variations in this kind of systems, which were normalized by randomization in the experimental design. With this in mind, we alternated position of lung slices from each donor in-between experiments, so we are confident in that we obtained reliable and comparable results, as highlighted by the data obtained from repopulated DLS and PCLS in culture.

As concerns the ECM environment, it showed to have a significant impact on cell function regarding the studied parameters in repopulated DLS, highlighting the importance of a methodology that accounts for this aspect of the *in vivo* milieu when studying mechanical stimuli. Our system showed compatibility with *in vitro* culture of both PCLS and repopulated lung slices using physiologically relevant stretch settings. Our PCLS data did not show any negative effects on either viability or tissue morphology and had low intra- and inter-experimental variability. This validates our system as a reliable way to introduce a physiomimetic stretch in complex lung models, and makes it comparable to other previously described devices (Davidovich et al., 2012; Lavoie et al., 2012; Mondoñedo et al., 2020).

Interestingly, cyclic stretch induced an increased expression of surfactant protein B (SFTPB) gene in epithelial cells after 1 day of culture in DLS, which is in line with other studies on alveolar epithelial cells (AEC) and H441 cells cultured under stretch in 2D (Sanchez-Esteban et al., 1998; Arold et al., 2009). As surfactant protein B facilitates breathing by lowering surface tension, a positive regulation in response to increased strain would be in line with its physiological function (Wert et al., 2009). Moreover, SFTPB production is believed to depend on the presence of a functional epithelial layer, which would indicate that the stretch movement did not significantly interfere with this process (Fehrholz et al., 2017). The reduction of SFTPB expression after 4 days suggests an earlier peak effect, similar to what has been found in other studies where the authors observed temporal changes in the expression of ventilator-induced lung injury markers over time (Dolinay et al., 2017). Therefore, our results suggest that our stretch device induced a physiologically relevant response to the mechanical stimulation in H441 epithelial cells cultured in a lung-derived ECM.

Previous data on mechanostimulation of H441 cells in monolayer culture have shown a mitogenic response (Chess et al., 2000), which was not seen in our data, however, they used both a higher frequency of 1 Hz and a larger amplitude with about 20% elongation of the cells. Another important difference is the influence of the ECM scaffold employed in our study, where the proportion of proliferative cells decreased from day 1 to day 4, regardless of stretching, possibly due to spatial constraints as the available spaces within the 3D structures were filled. The high proportion of proliferative cells at day 1 would make an increase in cell number to day 4 conceivable, even without a concurrent change in metabolism as these parameters are known to show poor correlation in cancerous cells (Quent et al., 2010).

As a well-known downstream effector of cellular mechanoresponses, transcriptomic regulator YAP1 is one of several important factors controlling cell proliferation and differentiation (Cai et al., 2021). Surprisingly, we did not find significant differences in the levels of active nuclear YAP1 between cyclic stretching and the static control. As not only the nature of the stretching movement, but also the presence of an ECM and cell-cell contacts may modify YAP1 activity, the interpretation of this result is challenging. Indeed, it has been described that mechanical stretch and ECM stiffness share mechanosensing pathways to regulate YAP1, and high cell densities have shown to inactivate YAP1 through different mechanisms (Cai et al., 2021). Moreover, a recent study showed that a cyclic stretch frequency below 0.25 Hz, similar to the one used in this work, did not promote YAP1 activation *in vitro* (Andreu et al., 2021). However, this comparison is limited, since the stretch device and the substrate thickness are different compared to the ones used here. Furthermore, the results were obtained after a short (1 h) stretch, and the type of cyclic stretch did not follow a sinusoidal curve as in our case.

As we hypothesised that mechanical stimuli could affect the synthesis of ECM components, we examined gene expression for two key basement membrane components in alveoli, laminin α-5 and collagen type IV, as well as the gene for a collagen type III that has been shown to be upregulated in response to noxious mechanical stimuli in the setting of ventilator-induced lung injury (Timpl, 1989; Pierce et al., 1998; Pelosi and Rocco, 2008). However, cyclic stretch did not alter the expression of these three genes, but there was a clear effect of the 3D microenvironment of the DLS compared to the regular TCP for collagen type IV and laminin α-5, resulting in a lower expression of these proteins. Both collagen IV and laminin are retained after decellularization of the lung slices (Rosmark et al., 2018). As these proteins are involved in cell adhesion, the presence of a pre-formed basement membrane in the DLS might decrease the stimuli for the repopulating cells to produce these proteins as compared to culture on tissue culture plastic. Some support of such negative feedback loops exists from studies of the regulation of laminin secretion (Hopkinson et al., 2008).

The use of *in vitro* devices to simulate physiologically relevant mechanical stimuli is currently fraught with some important limitations. The first relates to the limited understanding regarding the nature of the mechanical distension of alveolar cells that occur in the lungs. As it is inherently difficult to measure alveolar wall distention *in vivo*, most of the available information comes from studies on animal lungs removed from the chest cavity, which means that the forces normally expanding the lungs are removed or altered. The linear distension of alveolar walls was estimated to be in the range of ∼0%–5% for tidal breathing and ∼15%–40% going from residual volume to total lung capacity, in a review of available studies (Roan and Waters, 2011). Based on the studies summarized in this review, an estimate of surface area increase could be around double that for the linear distension. This is well in line with the 10% increase in stretch used in our study in attempting to mimic tidal breathing. A limitation of the applied technique is that the mechanical forces are applied in only two dimensions, as opposed to the three dimensions in the *in vivo* condition, so the forces might not be equally distributed throughout the lung slices (Dassow et al., 2010). While other piston-based stretch devices have been developed, most of them are used in 2D cultures (Kim, 2020) and they are not easily applicable to 3D structures or slices like the ones used here, i.e., DLS and PCLS. As regards our system, another possibly contributing factor to the limited effects in the readouts from the H441 cell line is related to their growth pattern, which in our case resulted in a large proportion of cells growing on top of other cells, and not as monolayers attached to the ECM, likely leading to a less efficient transfer of mechanical forces to these cells. Future studies should focus on the use of other cell types that mimic more closely the physiological situation, like alveolar type I cells that make up around 90% of the alveolar epithelial surface, a co-culture of cell lines that represent both alveolar type I and type II populations in the alveoli, or primary epithelial cells, in general.

Herein, we showed a proof-of-concept study of the use of a novel stretch device for 3D *in vitro* models including repopulated lung scaffolds and PCLS. This is, to our knowledge, a unique device that is easily compatible with slices or scaffolds that simulate the lung ECM. The precise control of the movement allowed for a cyclic stretch mimicking tidal breathing applied to epithelial cells cultured in a human lung derived ECM microenvironment and PCLS. Our data highlight the importance of the ECM microenvironment, as the culture in lung derived ECM had more impact than the dynamic mechanical stimuli applied on several of the cellular activities evaluated. Nevertheless, stretch of the lung slices promoted changes in the expression of SFTPB, which suggests that our stretch device and methodology supply physiologically relevant mechanical stimuli. Finally, PCLS were cultured successfully, supporting cell viability in a reproducible manner. Therefore, we conclude that the stretch device developed in this work shows promise as a tool for studies of cell and tissue function in a system integrating both a relevant ECM milieu and physiological or pathological mechanical stimuli.

## Data Availability

The raw data supporting the conclusion of this article will be made available by the authors upon request, without undue reservation.
