# Supplementary material for: A tunable physiomimetic stretch system evaluated with precision cut lung slices and recellularized human lung scaffolds
Source: Front Bioeng Biotechnol. 2022 Oct 3;10:995460. doi: 10.3389/fbioe.2022.995460 (PMC9574011; doi:10.3389/fbioe.2022.995460)
Supplement: Supplementary file 1 [file DataSheet1.PDF]

Supplementary Material

1 Supplementary Figures and Tables

1.1 Supplementary Figures

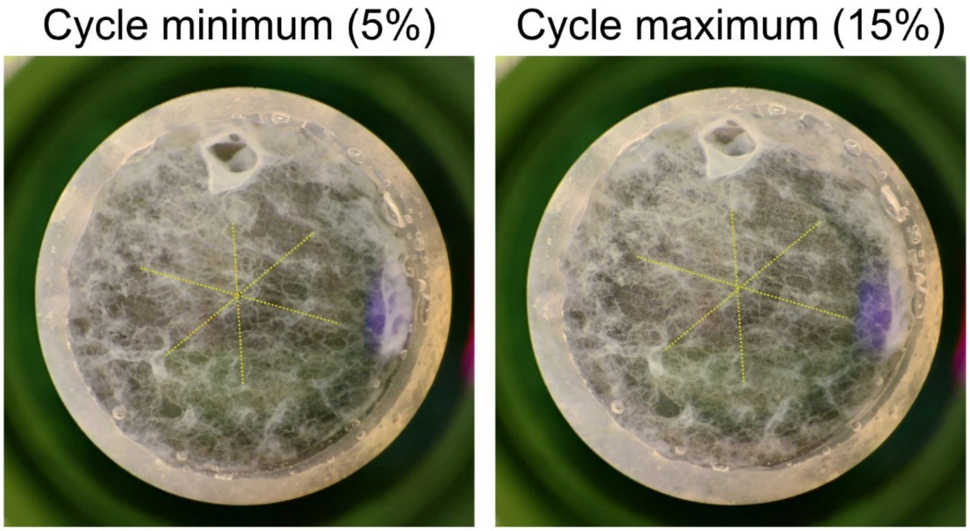

Supplementary Figure 1. Measurement of the stretch. Images showing the lines drawn to measure the amount of stretch produced by the device. The lines are used as diameters of a circle, which area is used for comparison between different theoretical stretching values. Representative 5 and 15% stretch images obtained with a stereomicroscope are shown as an example. The same strategy was used for all the measured images.

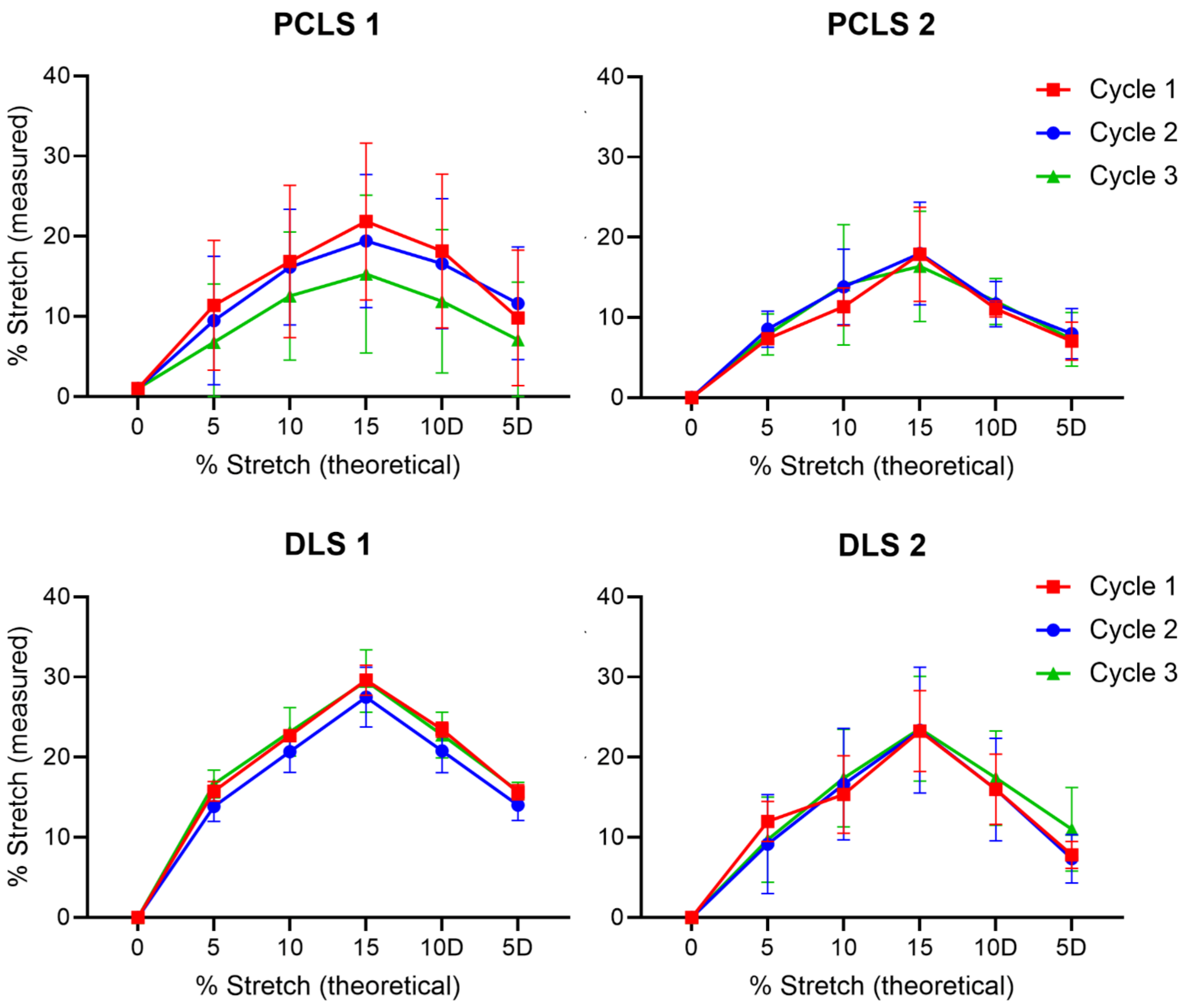

Supplementary Figure 2. Calibration of the stretch at different theoretical values. Two PCLS and two DLS were used to measure the stretch at different theoretical values, namely 0.2, 5, 10, and 15% stretch, both increasing and decreasing (10D and 5D) in a pseudocycle. This means that the measurement was done on images taken after stopping at each stretch value, finally representing the cycle. Graphs represent the measured stretch value against the theoretical one. Three consecutive cycles are represented in red (cycle 1), blue (cycle 2) and green (cycle 3), with datapoints represented with squares, circles and triangles, respectively.

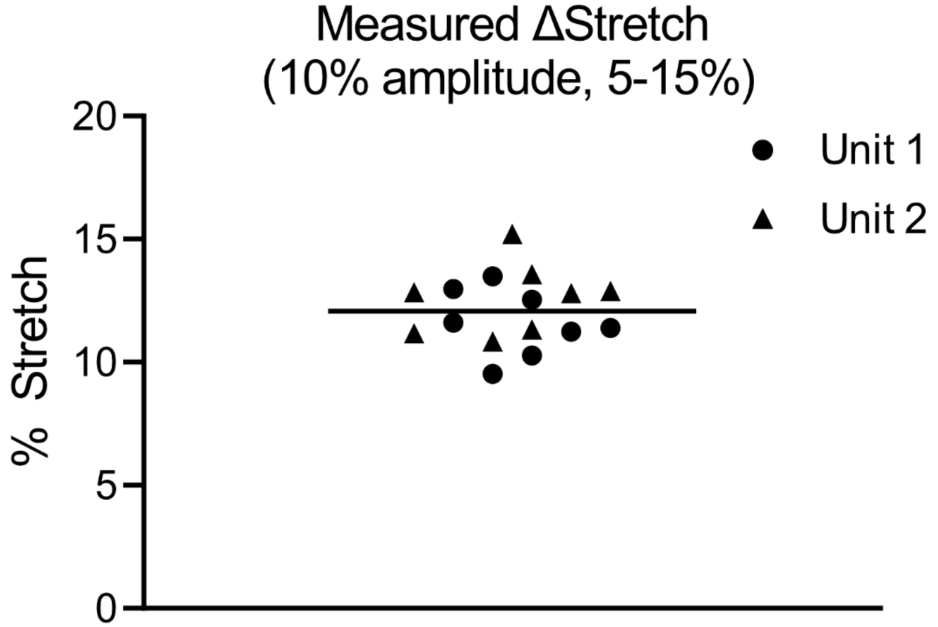

Supplementary Figure 3. Well-to-well stretch measurement. The percentage of stretch was measured in the eight wells of the two stretch device units available in our laboratory. One DLS was used per device. The theoretical stretch amplitude was 10% (from 5% baseline to 15% maximum stretch), as used in the experiments involving repopulated DLS and PCLS. All the datapoints are shown, with the median as a line. Stretch measurements for Unit 1 are represented as circles, while those for Unit 2 are shown as triangles.

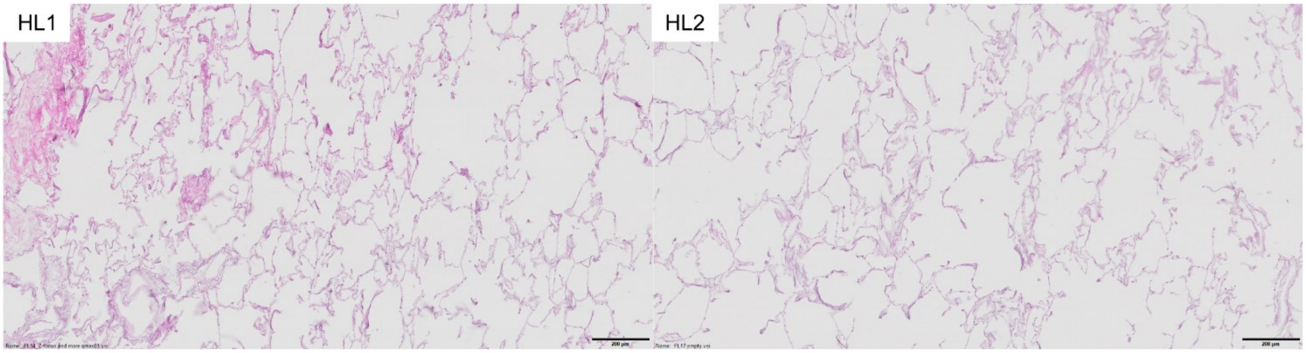

Supplementary Figure 4. Decellularized lung slices (DLS). Representative images of hematoxylin and eosin staining of DLS from two different healthy lung donors, HL1 and HL2, after decellularization. No cell nuclei were observed. Scale bar: 200 µm.

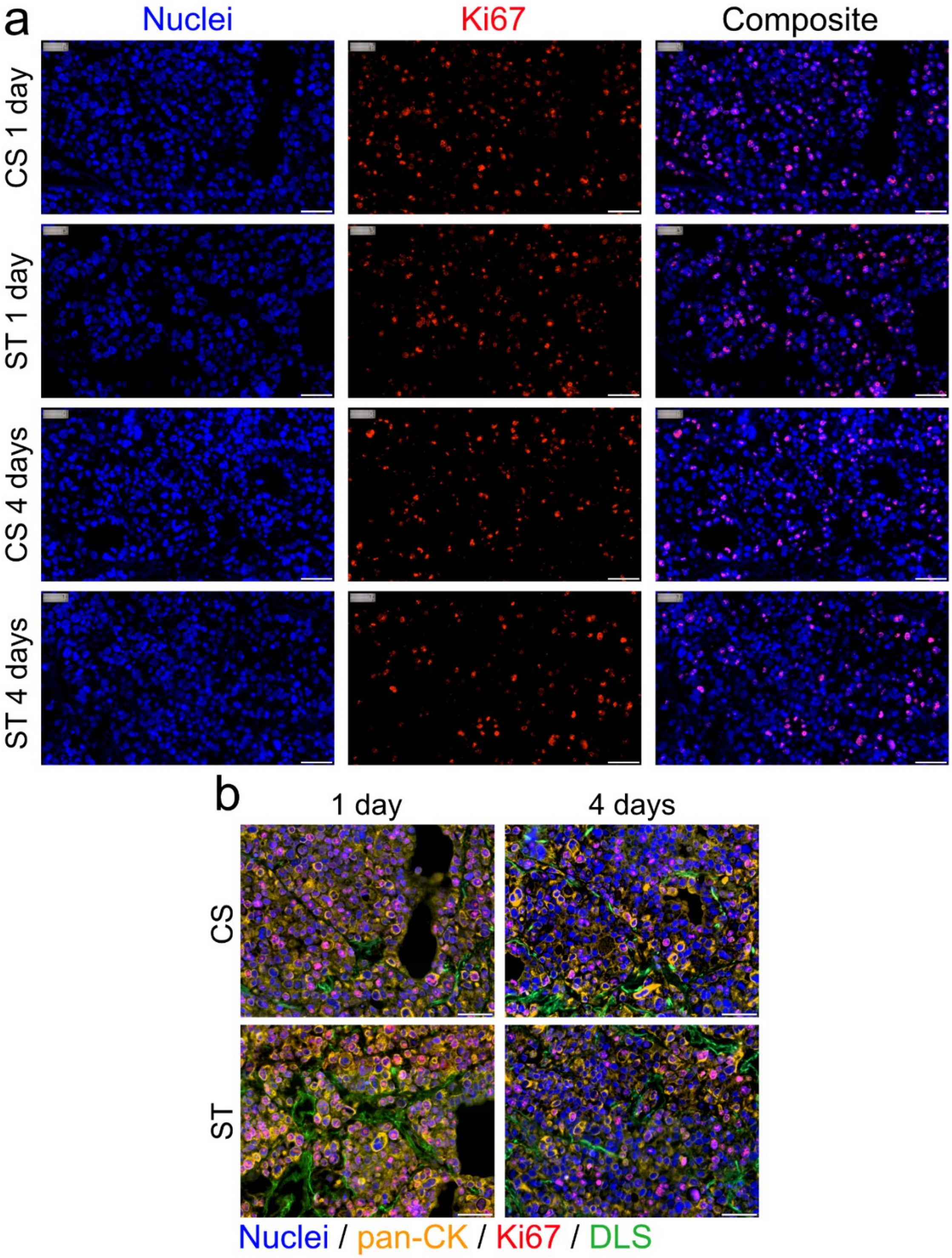

Supplementary Figure 5. Ki67 staining of recellularized DLS. (**A**) Representative images of DLS recellularized with H441 cells after 1 and 4 days for CS and ST conditions, stained for nuclei (DAPI) and Ki67 (in red). (**B**) Composite images of Ki67 staining for CS and ST conditions after 1 and 4 days of culture, including the staining of pan-cytokeratin (pan-CK, in yellow) and the autofluorescence of the DLS (in green). Scale bar: 100 µm.

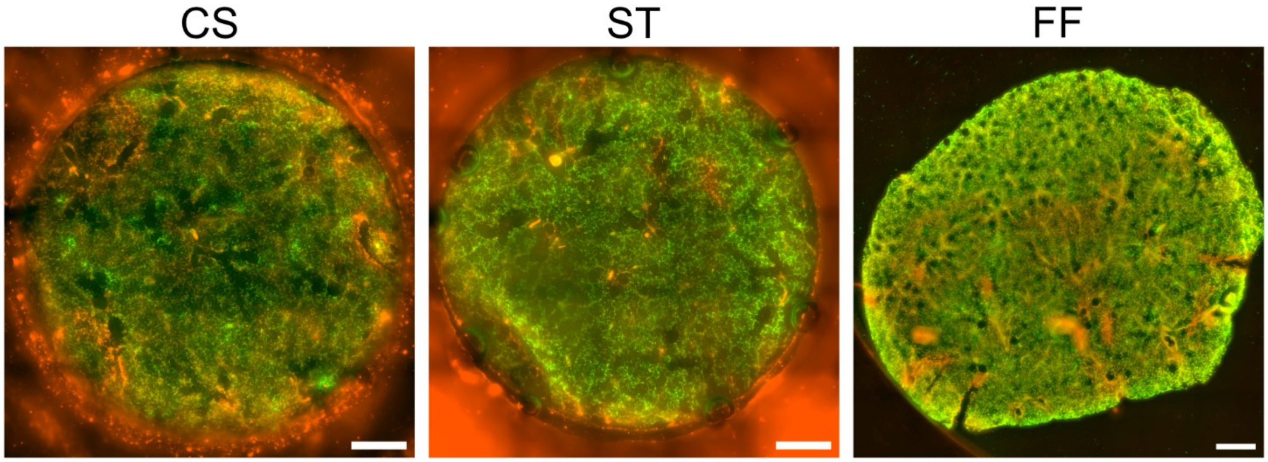

Supplementary Figure 6. LIVE/DEAD staining of rat PCLS. Representative maximum intensity Z-projection images of rat PCLS stained with a LIVE/DEAD staining to determine the effect of the stretch (CS) or the mounting (ST) on cell viability, compared to free-floating (FF) slices after 24 h of culture. Living cells are represented in green, while dead ones appear in red. Autofluorescence is observed in red around the tissue mounted in the stretch device well in CS and ST. Scale bar: 1 mm.

1.2 Supplementary Tables

Supplementary Table 1. Overview tissue donors for DLS. DLS: decellularized lung slice; HL: healthy lung donor.

Designation	Reason for surgery	Age (years)	Sex	Smoking status
HL1	organ donor	62	male	former
HL2	organ donor	66	male	former
HL3	organ donor	68	male	never
HL4	organ donor	26	male	former
HL5	organ donor	66	male	current

Supplementary Table 2. QuantiTect primers used in the qPCR analysis.

Primer name	Target gene	Target gene acronym	Catalog id	Company
Hs_PPIA_1_SG	Peptidylprolyl isomerase A	PPIA	QT00052311	Qiagen
Hs_COL3A1_1_SG	Collagen III, alpha 1 chain	COL3A1	QT00058233	Qiagen
Hs_COL4A3BP_1_SG	Collagen IV, alpha 3 chain	COL4A3	QT00032123	Qiagen
Hs_LAMA5_1_SG	Laminin alpha 5	LAMA5	QT00077819	Qiagen
Hs_SFTPB_1_SG	Surfactant protein B	SFTPB	QT00082404	Qiagen

Supplementary Table 3. Antibodies used for IF stainings.

Antigen	Company	Catalog id	Isotype	Dilution	Conjugation
active YAP1	Abcam	ab205270	Rabbit IgG	1:500	-
Ki67	Abcam	ab15580	Rabbit IgG	1:500	-
pan-Cytokeratin	Abcam	ab27988	Mouse IgG1	1:100	-
Rabbit IgG	Thermo Fisher	A32733	Goat IgG	1:200	Alexa Fluor Plus 647
Mouse IgG	Thermo Fisher	A31570	Donkey IgG	1:200	Alexa Fluor 555
